# Effects of upper limb robot-assisted therapy on motor recovery in subacute stroke patients

**DOI:** 10.1186/1743-0003-11-104

**Published:** 2014-06-19

**Authors:** Patrizio Sale, Marco Franceschini, Stefano Mazzoleni, Enzo Palma, Maurizio Agosti, Federico Posteraro

**Affiliations:** 1 Department of Neurorehabilitation, IRCCS San Raffaele Pisana, Rome, Italy; 2 The BioRobotics Institute, Scuola Superiore Sant’Anna, Pontedera, Italy; 3 Laboratory of Rehabilitation Bioengineering, Auxilium Vitae Rehabilitation Center, Volterra, Italy; 4 Rehabilitation Hospital Parma, Parma, Italy; 5 Rehabilitation Department, Versilia Hospital-AUSL12, Camaiore, Italy

**Keywords:** Stroke, Robotics, Rehabilitation, Upper limb

## Abstract

**Background and purpose:**

There is little evidence available on the use of robot-assisted therapy in subacute stroke patients. A randomized controlled trial was carried out to evaluate the short-time efficacy of intensive robot-assisted therapy compared to usual physical therapy performed in the early phase after stroke onset.

**Methods:**

Fifty-three subacute stroke patients at their first-ever stroke were enrolled 30 ± 7 days after the acute event and randomized into two groups, both exposed to standard therapy. Additional 30 sessions of robot-assisted therapy were provided to the Experimental Group. Additional 30 sessions of usual therapy were provided to the Control Group.

The following impairment evaluations were performed at the beginning (T0), after 15 sessions (T1), and at the end of the treatment (T2): Fugl-Meyer Assessment Scale (FM), Modified Ashworth Scale-Shoulder (MAS-S), Modified Ashworth Scale-Elbow (MAS-E), Total Passive Range of Motion-Shoulder/Elbow (pROM), and Motricity Index (MI).

**Results:**

Evidence of significant improvements in MAS-S (p = 0.004), MAS-E (p = 0.018) and pROM (p < 0.0001) was found in the Experimental Group. Significant improvement was demonstrated in both Experimental and Control Group in FM (EG: p < 0.0001, CG: p < 0.0001) and MI (EG: p < 0.0001, CG: p < 0.0001), with an higher improvement in the Experimental Group.

**Conclusions:**

Robot-assisted upper limb rehabilitation treatment can contribute to increasing motor recovery in subacute stroke patients. Focusing on the early phase of stroke recovery has a high potential impact in clinical practice.

## Introduction

A progressive decrease in stroke mortality has been observed over the past years in western Countries together with a subsequent increase in survivors with residual impairments and disabilities that require assistance [1]. The most frequent impairment caused by stroke is the restriction of motor activity, which reduces muscle movement and mobility [2], although stroke may also lead to sensory and cognitive impairment as well. Moreover the ability to carry out the activities of daily living (ADLs) in an autonomous way and to be engaged in social and community participation is strongly reduced [3]. Up to 85% of stroke patients experience hemiparesis immediately after stroke, while a number of survivors between 55% and 75% continue to experience motor deficits, together with a diminished quality of life [4].

The recovery of upper and lower limb function after stroke injuries is a complex process and requires multidisciplinary and multi-factorial approaches However, upper limb functional recovery requires long physical rehabilitation treatment in order to recover maximum independence and the highest quality of life possible. Different intensive methods can be used to achieve these results but no clear evidence for the best treatment is yet available [5]. Intensive task-oriented training could contribute to achieving upper and lower limb impairment reduction even if this process is driven mainly by adaptive strategies that provide a compensation of impaired motor activity [6-8].

Scientific evidence shows that a multi-factorial approach and high intensity therapy are able to improve motor recovery of upper limbs in stroke rehabilitation [9-12]. Passive [13-15] and active upper limb movements [16-18] seem to increase motor recovery, due to effects on somatosensory input, motor planning, soft tissue properties and spasticity.

A number of robotic devices, which have proven to improve arm motor performance, at least in chronic stroke patients, have been developed over the past decade to deliver targeted sensorimotor training to patients with neurological diseases such as stroke.

Different robot-assisted rehabilitation approaches have been provided in chronic stroke patients in order to improve arm function, including mono-lateral versus bilateral training and/or proximal versus distal approaches [19]. Kwakkel and colleagues conducted a meta-analysis of RCTs and reported a significant improvement in upper limb motor impairment, whereas they did not find any significant changes in ADLs using upper limb robot-assisted treatment in chronic stroke patients [20].

Lo and colleagues demonstrated that the robotic system for shoulder/elbow rehabilitation on chronic post-stroke patients did not significantly improve motor performance after 12 weeks compared to usual care or intensive therapy. Nevertheless, secondary analyses showed that the robot-assisted therapy compared to usual care rather than intensive therapy improved outcomes over 36 weeks [21].

On the contrary, definitive scientific evidence on the use of robotic upper limb treatment has not been found to date in subacute stroke patients [22-24]. Indeed, only 3 randomized controlled trials (RCTs) with different methodological approaches have been carried out [18]: the results showed that upper limb robotic training during the subacute phase can contribute to improving functional abilities more than chronic phase training without any comparison with usual rehabilitative treatment. Moreover, two RCTs were carried out to demonstrate the effectiveness of distal upper limb robotic therapy in subacute stroke patients without any significant result [25,26].

The objective of the two-center randomized controlled observer-blind trial presented in this article is to evaluate the effects of robot-assisted therapy on upper limb body function (impairment) compared to usual intensive physical therapy, at the early phase after stroke onset. An analysis of clinical outcome measures at the middle and at the end of the treatment is also provided.

## Methods

### Participants

Eligible hemiparetic stroke survivors from San Raffaele Pisana, Rome, Italy and Auxilium Vitae Rehabilitation Centre, Volterra, Italy were recruited. The study included only subacute stroke patients at their first-ever stroke enrolled 30 ± 7 days after the acute event with ischemic lesions and hemorrhagic forms only. The diagnoses were confirmed with CT scan and/or MRI exam.

Inclusion criteria for both groups were: (a) first acute event of cerebrovascular stroke; (b) unilateral paresis; (c) ability to understand and follow simple instructions; (d) ability to remain in a sitting posture. The following exclusion criteria were identified: (e) bilateral impairment; (f) severe sensory deficits in the paretic upper limb; (g) cognitive impairment or behavioral dysfunction that would influence the ability to comprehend or perform the experiment; (h) refusal or inability to provide informed consent; (i) other current severe medical problems.

The local Ethical Committee of both centers approved the study. All patients gave informed consent to the investigation.

### Apparatus

**Figure 1:**
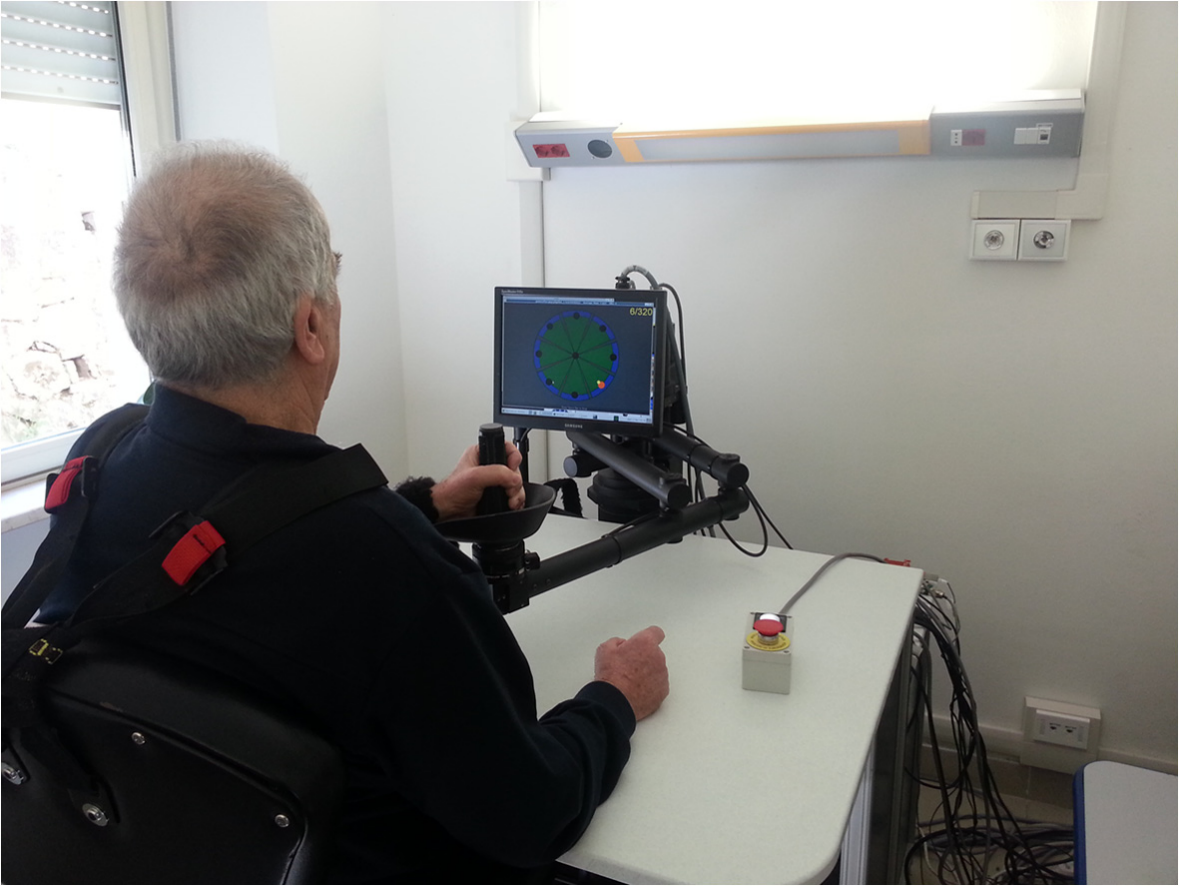
Experimental setup.

The MIT-MANUS/InMotion2 (Interactive Motion Technologies, Inc., Watertown, MA, USA) (IM2) is a robotic device designed for the rehabilitation of shoulder and elbow segments [27] (Figure 1). The IM2 has two translational degrees of freedom (DoFs): shoulder abduction-adduction and elbow flexion-extension. The robotic system supports the execution of reaching movements in the horizontal plane through an assist-as-needed control strategy. In particular, an impedance-based control strategy is implemented: the robot assists the motion of the upper limb during the execution of planar trajectories according to a mass-spring-damper model which mimics the interaction with the therapist through control parameters. Based on the specific performance of each patient, these parameters are adjusted during the therapy using data recorded by the robot sensors (force sensors, encoders, tachometers). Physical quantities, such as position, velocity and applied forces, were recorded at the robot end-effector.

The device is designed to have a low intrinsic end-point impedance (i.e., to be backdriveable, execute movements without constraint and offer minimal resistance). A visual feedback based on the patient’s motor performance is provided every 80 movements. The paretic arm is placed in a custom-made arm support (available for both arms) fixed to the robot end-effector.

### Procedures

Fifty-three inpatients with first-ever ischemic mono-hemispheric stroke were randomly assigned to two groups, Experimental Group (EG) and Control Group (CG). The random allocation to treatment was concealed and based upon dedicated software. A Lehemer algorithm was applied to achieve a balanced allocation in the EG and CG groups. Therapists were randomly assigned to patients within each group using the same algorithm. The clinical assessments were carried out by blinded assessors at the beginning (T0), after 15 sessions (T1), and at the end of the treatment, after 30 sessions (T2).

**Figure 2:**
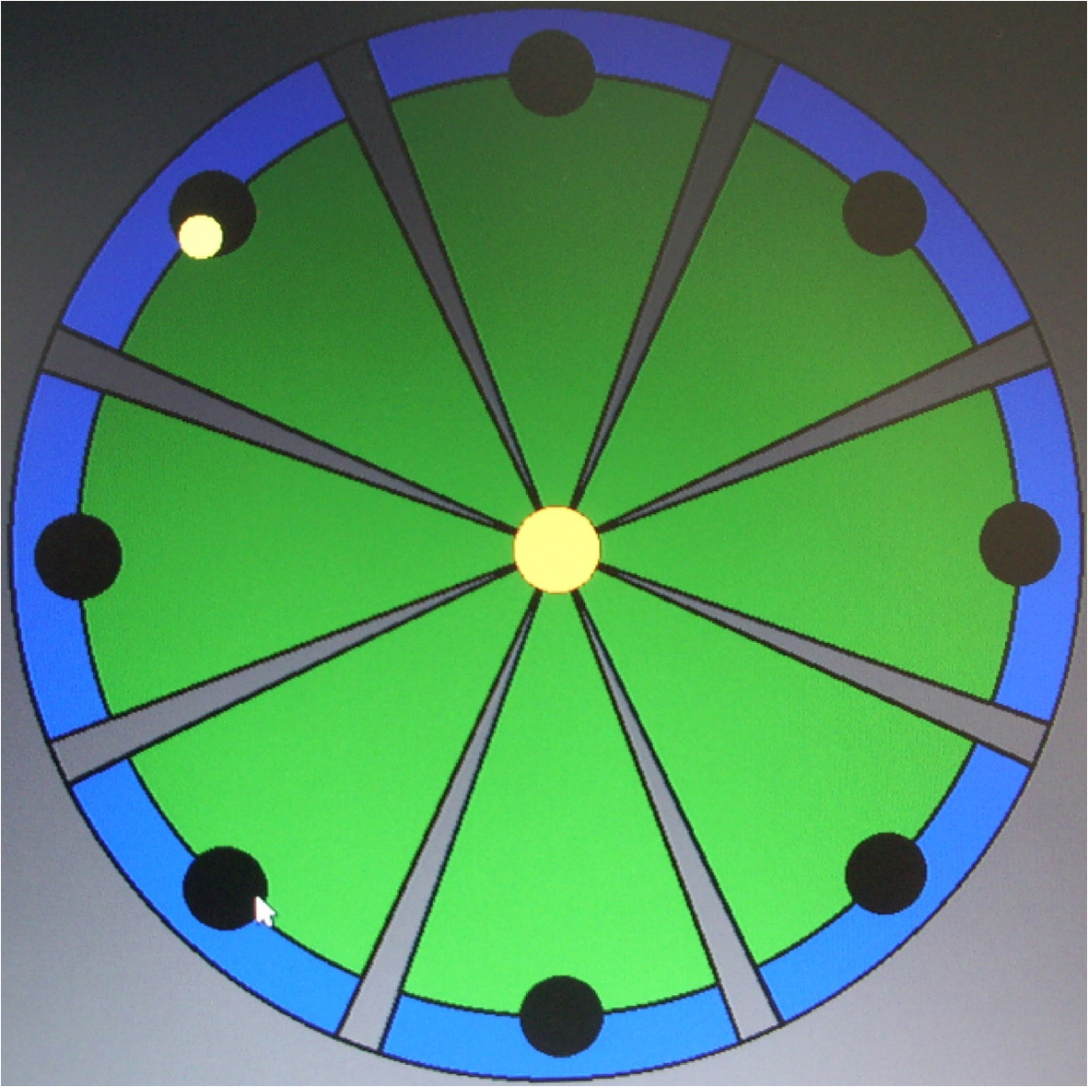
Clock-like rehabilitation scenario.

All subjects underwent inpatient rehabilitation, consisting of a daily 3-hour physiotherapy session, including both dexterity and gait training, according to individually tailored exercise scheduling. In addition to usual rehabilitation, eligible patients also received one daily session of either experimental or control treatment according to the random allocation procedure. Therapy was always performed under the supervision of a physical therapist.Each subject in the EG was asked to perform 30 sessions (5 days a week for 6 weeks) of goal-directed, planar reaching tasks, which emphasized shoulder and elbow movements, moving from the center target to each of the 8 peripheral targets, equally spaced on a 0.14 m radius circumference around a center target (Figure 2), using the IM2 robot. In each session, patients were asked to perform a series of 16 unassisted clockwise repetitions to each target, followed by 3 series of 320 assisted clockwise repetitions. At the end of each adaptive series, the patient was asked to perform an additional series of 16 unassisted clockwise movements. After 45 minutes the session was stopped.

Each subject in CG received 30 sessions (5 days a week for 6 weeks) of conventional rehabilitative treatment, matching robot-assisted therapy of the same duration, such as assisted stretching, shoulder and arm exercises, and functional reaching tasks provided by experienced physiotherapists [23]. Every missed session was caught up and subjects who were not able to catch up sessions or who interrupted the treatment for more than 3 consecutive days were excluded from the study.

### Outcome measures

Clinical outcomes were assessed using valid and reliable stroke tools that include all International Classification of Functioning, Disability and Health levels.

The upper limb section of the Fugl-Meyer Test (FM) [28], which is a global evaluation scale for impairment in stroke patients, and the Modified Ashworth Scale-Shoulder (MAS-S) and Elbow (MAS-E), as outcome measure assessing spasticity [29], were selected as primary outcomes.

The secondary outcomes were Passive Range of Motion (pROM), as the sum of shoulder and elbow movements (shoulder flexion/extension, abduction, intra/extrarotation and elbow extension), to assess the joints excursion which could be considered correlated to spasticity [30], and Motricity Index (MI) that globally assesses impairment in stroke patients [31]. Trained professionals, who were not involved in the research and were blind to group allocation, performed all the assessments.

### Sample calculation and statistical analysis

A pre-study power calculation estimated that 25 participants would provide 80% power with 5% alpha to detect a difference of 9 ± 10 points in FM between the two groups. To assess the homogeneity of the two groups by age, length of illness and outcome measures, we used Mann–Whitney *U* Test independent samples to compare median scores, whilst the Fisher’s Exact Test was used for frequencies.

With regard to interval variables, the 2 groups (EG and CG) were compared at the intermediate phase (difference T1 - T0) and at the end of the treatment (difference T2 – T1) using a 2 × 2 repeated-measures analysis of variance (two-way ANOVA). In the presence of statistically significant effects, post-hoc comparisons were performed by comparing the change between T0 and T1, and between T0 and T2 using the Wilcoxon signed rank test.

The alpha level for significance was set at p < 0.05. Statistical analysis was carried out using the SPSS version 19.0 (SPSS Inc, Chicago, Illinois, USA).

## Results

**Figure 3:**
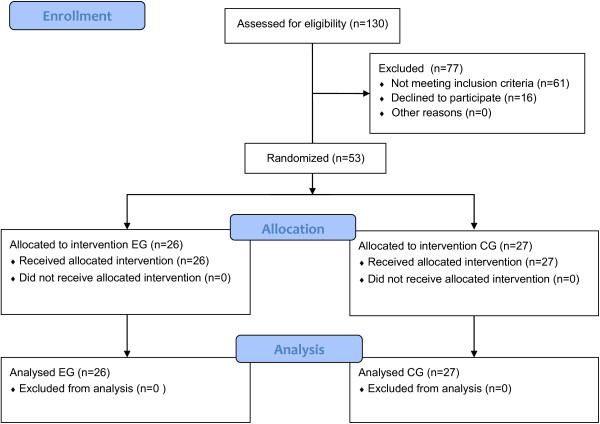
**Study CONSORT flow diagram (Abbreviation: EG, Experimental Group, CG, Control Group).**

130 patients were screened, 53 of whom satisfied the inclusion criteria and were randomly assigned to the groups as follows: 26 to the EG and 27 to the CG. No dropouts were observed during treatment within each group: all subjects completed the study protocol (Figure 3). Despite higher FM and MI values in the EG at T0 than in the CG, the distribution of the study subjects (N = 53) by age, gender, etiology, lesion side and outcome measures did not show any significant difference between the two groups (Table 1), as shown by the Mann–Whitney *U* test and Fisher’s Exact Test which did not detect any statistical significant differences at T0 between the two groups.

**Table 1:** **Patients**’ **characteristics at baseline** (**n** = **53**)

**Characteristics**	**Experimental Group**	**Control Group**	**Exact Sig. (2-sided)**
	**n (%)**		
Subject	26 (49%)	27 (51%)	
Dropouts	0 (0%)	0 (0%)	
Compliants	26 (100%)	27 (100%)	
Gender			1.000^a^
*Female*	11 (42%)	11 (41%)	
*Male*	15 (58%)	16 (59%)	
Etiology			.100^a^
*Hemorrhagic*	1 (4%)	6 (22%)	
*Ischemic*	25 (96%)	21 (78%)	
Lesion Side			.412^a^
*Right*	10 (38%)	14 (52%)	
*Left*	16 (62%)	13 (48%)	
	**Mean (SD) - Median [25th-75th percentiles]**		
Age, years	67.7 (14.2) - 72.0 [65.8-77.0]	67.7 (14.2) - 73.0 [69.0-78.0]	.294^b^
CMSA	3.0 (1.2) - 2.0 [2.0-4.0]	3.0 (1.2) - 2.0 [2.0-3.0]	.053^b^

^a^Fisher’s Exact Test.

^b^Mann–Whitney Exact Test.

*Abbreviations*: *SD* standard deviation, *CMSA* Chedoke-McMaster Stroke Assessment.

As shown in Table 2, the primary outcome analysis using the two-way ANOVA test showed statistically significant improvements in MAS-S (p = 0.004) and MAS-E (p = 0.018) in the EG.

**Table 2:** **Clinical outcome measures at T0**, **T1 and T2**

	**Baseline**	**After 15 sessions**	**End of treatment**	**ANOVA**	**T1**-**T0**	**T2**-**T0**
	***CG*: *n*** **=** ***27*;**	***CG*: *n*** **=** ***27*;**	***CG*: *n*** **=** ***27*;**			
	***EG*: *n*** **=** ***26***	***EG*: *n*** **=** ***26***	***EG*: *n*** **=** ***26***			
	**T0**	**T1**	**T2**	***p***-***value***	***p***-***value***	***p***-***value***
	***(Mean ± Sd)***	***(Mean ± Sd)***	***(Mean ± Sd)***			
**Motricity index**						
*CG*	30.30 ± 33.38	35.78 ± 34.20	39.56 ± 35.10	<*0.0001*	*0.008*	*0.002*
*EG*	43.88 ± 24.77	53.77 ± 25.80	57.77 ± 24.22	<*0.0001*	<*0.0001*	<*0.0001*
**Fugl-Meyer**						
*CG*	20.33 ± 16.01	22.30 ± 16.52	23.96 ± 17.51	<*0.0001*	*0.001*	*0.001*
*EG*	26.81 ± 11.43	34.15 ± 12.49	35.46 ± 12.24	<*0.0001*	<*0.0001*	<*0.0001*
**pROM**						
*CG*	791.48 ± 75.3	805.22 ± 66.2	792.59 ± 83.0	*NS*	*NS*	*NS*
*EG*	755.0 ± 105.1	787.31 ± 98.1	809.04 ± 90.5	<*0.0001*	<*0.0001*	<*0.0001*
**MAS-S**						
*CG*	1.19 ± 1.0	1.19 ± 1.14	1.15 ± 1.17	*NS*	*NS*	*NS*
*EG*	1.15 ± 1.16	0.81 ± 1.10	0.73 ± 1.08	*0.004*	*NS*	*0.030*
**MAS-E**						
*CG*	0.85 ± 0.91	0.85 ± 0.91	0.93 ± 0.96	*NS*	*NS*	*NS*
*EG*	1.12 ± 1.07	0.92 ± 1.02	0.73 ± 0.96	*0.018*	*0.020*	*0.010*

A not statistically significant decreasing trend of MAS-S and increasing trend of MAS-E was found in the CG.

FM improved significantly in both groups (EG: p < 0.0001, CG: p < 0.0001). It is noteworthy that the statistical analysis of the change in FM between T0 and T1 and between T0 and T2 provided by a Wilcoxon signed rank test showed a greater improvement in the EG than the CG after the first 15 sessions (p < 0.0001 and p < 0.001, respectively).

The secondary outcome showed statistically significant improvements in pROM (p < 0.0001) and MI (p < 0.0001) in the EG (Table 2), whereas the CG showed statistically significant improvements in MI (p < 0.0001) and a not statistically significant increase in pROM. The statistical analysis of the change in pROM between T0 and T1 and between T0 and T2 showed an increase just after the first 15 sessions (p < 0.0001) in the EG whilst pROM did not change in the CG.

As regards MI, a greater improvement in the EG than the CG after the first 15 sessions (p < 0.0001 and p = 0.008, respectively) was observed.

## Discussion

This study presents the results of a RCT carried out with the aim of systematically evaluating the effects of robot-assisted proximal upper limb treatment in subacute stroke patients. The focus on the early phase of stroke recovery is a further innovative feature of this study and makes our research useful to clinical practice, in terms of potential applicability to the population of stroke patients and fostering to treat patients with moderate to severe upper limb impairment.

Previous studies on chronic stroke patients showed a significant decrease in shoulder and elbow motor impairment after the robot-assisted treatment [32,33] and different systematic reviews have explored the effects of intensive therapy on functional recovery [34,35], even if a consensus on this issue has not yet been reached, in particular as regards the effects on ADLs. Our previous experience on chronic stroke patients demonstrated that intensive robotic training can improve upper limb functional activity in terms of ROM and FM [36-38].

Our results show that intensive robot-assisted treatment in subacute stroke patients may significantly reduce motor impairment in the paretic upper limb, with a statistically significant change in both pROM and MAS after 15 sessions of robotic treatment.

At the end of the treatment (i.e., 30 sessions), FM and MI improved significantly in both groups, even if the improvement in FM was higher in the EG than in the CG after 15 sessions, confirming that intensive training provided by robotic device contributes to obtain better results than usual treatment at the early stage of rehabilitation [39].

Rehabilitation strategies for stroke patients currently are focused on high-intensity, repetitive finalized and task specific training, even if a standardized protocol for upper limb rehabilitation, including timing, intensity and frequency, has not yet been shared.

Moreover it is widely accepted that rehabilitation treatment should start as soon as possible after the acute event, although there is not enough evidence supporting this assumption [40,41] and it remains still unclear i) the optimal overall duration of the intensive treatment stage and ii) if the use of technological devices may shorten it.

Spasticity usually appears days to weeks after the acute event and, although it may improve or disappear in some individuals, often becomes a permanent impairment as shown by electromyography studies which demonstrates that the increase in muscle tone reaches its maximum values between 1 and 3 months after stroke [42-44].

In our study statistical analysis on pROM and MAS shows that spasticity significantly decreased in the EG rather than in the CG, demonstrating positive effects of the robot-assisted treatment delivered in the early phase of rehabilitation in subacute stroke patients without any adverse events thus suggesting that the use of upper limb robotic treatment at the initial stage of stroke rehabilitation should be provided.

A limitation of this study relies on the use of outcome measures focused on the changes in the impairment level only and not on the changes in ADLs representing the final objective of the rehabilitation process.

However as the aim of the study was to assess (i) the effects on upper limb motor recovery and (ii) to compare the effectiveness of robotic therapy and usual care, clinical outcome measures exploring only body functions as referred in the International Classification of Functioning, Disability and Health (ICF) were used [45].

## Conclusions

Our results show that after 15 sessions of intensive robot-assisted rehabilitation therapy, moderate-to-severe upper-limb impaired stroke survivors improved more than those who received intensive traditional therapy. This finding was also confirmed after 30 sessions, although FM and MI significantly improved in both groups at the end of the treatment, showing an advantage in robot-assisted therapy only in the early stage of rehabilitation training.

Despite robotic rehabilitation is more frequently delivered to chronic stroke patients, where effectiveness is more associated with the intensity rather than with the specificity of the robotic approach, our results show that robot-assisted treatment provided in the subacute phase is able to effectively improve motor performance of the upper limb in a shorter time compared to usual intensive physiotherapy, thus accelerating the trend of motor recovery.

Moreover, our results show that an intensive active upper limb movement training in the rehabilitation of subacute stroke patients is able to reduce spasticity confuting the hypothesis that it could be responsible for an increased risk of spasticity development.

## Abbreviations

(FM): Fugl-Meyer Assessment Scale; (MAS-S): Modified Ashworth Scale-Shoulder; (MAS-E): Modified Ashworth Scale-Elbow; (pROM): Passive Range of Motion-Shoulder/Elbow; (MI): Motricity Index; (EG): Experimental Group; (CG): Control Group; (RCTs): Randomized controlled trials; (ADLs): Activities of daily living; (IM2): MIT-MANUS/InMotion2; (ANOVA): Analysis of variance.

## Competing interests

The authors declare that they have no competing interests.

## Authors’ contributions

The authors declare they have participated in the conception, design, analysis and interpretation of the results. They also drafted the manuscript and carried out a critical revision. In particular, FP and PS participated in the design of the study, acquired and analyzed the data, and drafted the manuscript. SM, EP and MF conceived the study, participated in its design and in the interpretation of data, and contributed to drafting the manuscript. PS, SM, and FP were involved in revising the manuscript. All authors read and approved the final manuscript. MA (biostatistician), PS and FP all had full access to the data.
